# Mega-analysis of association between obesity and cortical morphology in bipolar disorders: ENIGMA study in 2832 participants

**DOI:** 10.1017/S0033291723000223

**Published:** 2023-10

**Authors:** Sean R. McWhinney, Christoph Abé, Martin Alda, Francesco Benedetti, Erlend Bøen, Caterina del Mar Bonnin, Tiana Borgers, Katharina Brosch, Erick J. Canales-Rodríguez, Dara M. Cannon, Udo Dannlowski, Ana M. Diaz-Zuluaga, Lorielle M.F. Dietze, Torbjørn Elvsåshagen, Lisa T. Eyler, Janice M. Fullerton, Jose M. Goikolea, Janik Goltermann, Dominik Grotegerd, Bartholomeus C. M. Haarman, Tim Hahn, Fleur M. Howells, Martin Ingvar, Neda Jahanshad, Tilo T. J. Kircher, Axel Krug, Rayus T. Kuplicki, Mikael Landén, Hannah Lemke, Benny Liberg, Carlos Lopez-Jaramillo, Ulrik F. Malt, Fiona M. Martyn, Elena Mazza, Colm McDonald, Genevieve McPhilemy, Sandra Meier, Susanne Meinert, Tina Meller, Elisa M. T. Melloni, Philip B. Mitchell, Leila Nabulsi, Igor Nenadic, Nils Opel, Roel A. Ophoff, Bronwyn J. Overs, Julia-Katharina Pfarr, Julian A. Pineda-Zapata, Edith Pomarol-Clotet, Joaquim Raduà, Jonathan Repple, Maike Richter, Kai G. Ringwald, Gloria Roberts, Alex Ross, Raymond Salvador, Jonathan Savitz, Simon Schmitt, Peter R. Schofield, Kang Sim, Dan J. Stein, Frederike Stein, Henk S. Temmingh, Katharina Thiel, Sophia I. Thomopoulos, Neeltje E. M. van Haren, Cristian Vargas, Eduard Vieta, Annabel Vreeker, Lena Waltemate, Lakshmi N. Yatham, Christopher R. K. Ching, Ole A. Andreassen, Paul M. Thompson, Tomas Hajek, for the ENIGMA Bipolar Disorder Working Group

**Affiliations:** 1 Department of Psychiatry, Dalhousie University, Halifax, NS, Canada; 2 Department of Clinical Neuroscience, Karolinska Institutet, Stockholm, Sweden; 3 Vita-Salute San Raffaele University, Milan, Italy; 4 Division of Neuroscience, Psychiatry and Psychobiology Unit, IRCCS San Raffaele Scientific Institute, Milan, Italy; 5 Unit for Psychosomatics/CL Outpatient Clinic for Adults, Division of Mental Health and Addiction, Oslo University Hospital, Oslo, Norway; 6 Institut d'Investigacions Biomèdiques August Pi i Sunyer (IDIBAPS), Centro de Investigación Biomédica en Red de Salud Mental (CIBERSAM), Barcelona, Spain; 7 Department of Psychiatry, University of Münster, Münster, Germany; 8 Department of Psychiatry and Psychotherapy, Philipps-University Marburg, Marburg, Germany; 9 FIDMAG Germanes Hospitalàries Research Foundation, Barcelona, Spain; 10 Centre for Neuroimaging & Cognitive Genomics (NICOG), Clinical Neuroimaging Laboratory, NCBES Galway Neuroscience Centre, College of Medicine Nursing and Health Sciences, University of Galway, Galway, Ireland; 11 Research Group in Psychiatry GIPSI, Department of Psychiatry, Faculty of Medicine, Universidad de Antioquia, Medellín, Colombia; 12 Norwegian Centre for Mental Disorders Research (NORMENT), Institute of Clinical Medicine, University of Oslo and Division of Mental Health and Addiction, Oslo University Hospital, Oslo, Norway; 13 Department of Neurology, Division of Clinical Neuroscience, Oslo University Hospital, Oslo, Norway; 14 Institute of Clinical Medicine, University of Oslo, Oslo, Norway; 15 Department of Psychiatry, University of California, San Diego, La Jolla, CA, USA; 16 Desert-Pacific MIRECC, VA San Diego Healthcare, San Diego, CA, USA; 17 Neuroscience Research Australia, Randwick, NSW, Australia; 18 School of Medical Sciences, University of New South Wales, Sydney, NSW, Australia; 19 Department of Psychiatry, University Medical Center Groningen, University of Groningen, Groningen, The Netherlands; 20 Neuroscience Institute, University of Cape Town, Cape Town, South Africa; 21 Department of Psychiatry and Mental Health, University of Cape Town, Cape Town, South Africa; 22 Imaging Genetics Center, Mark and Mary Stevens Neuroimaging and Informatics Institute, Keck School of Medicine, University of Southern California, Marina del Rey, CA, USA; 23 Department of Psychiatry and Psychotherapy, University Hospital Bonn, Bonn, Germany; 24 Laureate Institute for Brain Research, Tulsa, OK, USA; 25 Department of Neuroscience and Physiology, Sahlgrenska Academy at Gothenburg University, Gothenburg, Sweden; 26 Department of Medical Epidemiology and Biostatistics, Karolinska Institutet, Stockholm, Sweden; 27 Department of Neurology, Institute of Clinical Medicine, University of Oslo, Oslo, Norway; 28 Institute for Translational Neuroscience, University of Münster, Münster, Germany; 29 Center for Mind, Brain and Behavior (CMBB), University of Marburg and Justus Liebig University Giessen, Marburg, Germany; 30 School of Psychiatry, University of New South Wales, Sydney, NSW, Australia; 31 Department of Psychiatry, Jena University Hospital/Friedrich-Schiller-University Jena, Jena, Germany; 32 UCLA Center for Neurobehavioral Genetics, Los Angeles, CA, USA; 33 Department of Psychiatry, Erasmus University Medical Center, Rotterdam, The Netherlands; 34 Research Group, Instituto de Alta Tecnología Médica, Ayudas diagnósticas SURA, Medellin, Colombia; 35 Institute of Psychiartry, King's College Londen, London, UK; 36 Department for Psychiatry, Psychosomatic Medicine and Psychotherapy, University Hospital Frankfurt, Goethe University, Frankfurt, Germany; 37 Oxley College of Health Sciences, The University of Tulsa, Tulsa, OK, USA; 38 West Region, Institute of Mental Health, Singapore, Singapore; 39 Yong Loo Lin School of Medicine, National University of Singapore, Singapore, Singapore; 40 South African MRC Unit on Risk & Resilience in Mental Disorders, University of Cape Town, Cape Town, South Africa; 41 Department of Child and Adolescents Psychiatry/Psychology, Erasmus MC Sophia Children's Hospital, Erasmus University Medical Center, Rotterdam, The Netherlands; 42 Department of Psychiatry, University Medical Center Utrecht Brain Center, University Medical Center Utrecht, Utrecht University, Utrecht, The Netherlands; 43 Erasmus School of Social and Behavioural Sciences Department of Psychology, Education & Child Studies Erasmus University, Rotterdam, The Netherlands; 44 University of British Columbia, Vancouver, BC, Canada; 45 National Institute of Mental Health, Klecany, Czech Republic

**Keywords:** Body mass index, obesity, bipolar disorders, cortical thickness, surface area, heterogeneity, lithium, antipsychotics

## Abstract

**Background:**

Obesity is highly prevalent and disabling, especially in individuals with severe mental illness including bipolar disorders (BD). The brain is a target organ for both obesity and BD. Yet, we do not understand how cortical brain alterations in BD and obesity interact.

**Methods:**

We obtained body mass index (BMI) and MRI-derived regional cortical thickness, surface area from 1231 BD and 1601 control individuals from 13 countries within the ENIGMA-BD Working Group. We jointly modeled the statistical effects of BD and BMI on brain structure using mixed effects and tested for interaction and mediation. We also investigated the impact of medications on the BMI-related associations.

**Results:**

BMI and BD additively impacted the structure of many of the same brain regions. Both BMI and BD were negatively associated with cortical thickness, but not surface area. In most regions the number of jointly used psychiatric medication classes remained associated with lower cortical thickness when controlling for BMI. In a single region, fusiform gyrus, about a third of the negative association between number of jointly used psychiatric medications and cortical thickness was mediated by association between the number of medications and higher BMI.

**Conclusions:**

We confirmed consistent associations between higher BMI and lower cortical thickness, but not surface area, across the cerebral mantle, in regions which were also associated with BD. Higher BMI in people with BD indicated more pronounced brain alterations. BMI is important for understanding the neuroanatomical changes in BD and the effects of psychiatric medications on the brain.

## Introduction

Obesity is the fifth leading cause of death globally and is one of the leading causes of disability (Di Angelantonio et al., 2016; GBD 2015 Obesity Collaborators et al., 2017; Nyberg et al., 2018). It is an even greater problem among people with severe mental illness (SMI) including bipolar disorders (BD). Based on meta-analysis of 120 studies, the pooled point prevalence of overweight and obesity in SMI was 60% and people with SMI had on average three times greater odds of obesity than the general population (Afzal et al., 2021). Aside from the impact of obesity on general health, obesity is commonly associated with structural brain alterations (Dekkers, Jansen, & Lamb, 2019; Fernández-Andújar, Morales-García, & García-Casares, 2021; García-García et al., 2019; Janowitz et al., 2015; Willette & Kapogiannis, 2015), and with an increased risk of cognitive impairment and dementia (Beydoun, Beydoun, & Wang, 2008; Pedditzi, Peters, & Beckett, 2016; Singh-Manoux et al., 2018; Tang et al., 2021). These issues may be particularly relevant in individuals who already have an increased risk of brain alterations (Hibar et al., 2018), cognitive impairment (Bora, Yucel, & Pantelis, 2009), and obesity (Vancampfort et al., 2015), such as people with BD. Individuals with BD and comorbid obesity face very specific challenges which require dedicated management and research efforts. However, very few studies up to date have investigated how the presence of obesity interacts with brain and cognitive changes in major psychiatric disorders.

Studying people with SMI and obesity could help identify preventable/treatable risk factors for neurostructural alterations, which may be associated with currently intractable psychiatric outcomes, including cognitive impairment. Indeed, we and others have shown that BD complicated by obesity-related metabolic alterations, specifically diabetes, is associated with lower psychosocial functioning (Hajek et al., 2005), higher rates of rapid cycling (Hajek et al., 2008), poor treatment response (Calkin et al., 2015), and worse psychiatric outcomes (Calkin et al., 2009). It could also explain why people with the same psychiatric diagnosis differ so markedly in their neurobiological/clinical outcomes, thus moving toward individualized medicine. Furthermore, weight gain is a common side effect of many psychiatric medications, which are also frequently associated with changes in brain structure. Therefore, we need to better understand the role obesity plays in the links between psychiatric disorders or medications and brain structure.

Previous studies in relatively small (76–112 participants) and highly selected groups [i.e. people with first episode of mania (Bond et al., 2014, 2011, 2019), adolescent BD participants (Islam, Metcalfe, MacIntosh, Korczak, & Goldstein, 2018), or offspring of people with BD (Mansur et al., 2018)] have suggested that obesity may be associated with brain alterations in BD, possibly with a stronger effect size or with some regional specificity compared to controls (Bond et al., 2014, 2011, 2019; Islam et al., 2018; Mansur et al., 2018). In a study including 2735 individuals (McWhinney et al., 2021a), we demonstrated that some of the most replicated subcortical brain alterations in BD, including larger ventricles, were to a large extent (up to 47%) mediated by obesity and that both BD and obesity were associated with similar subcortical alterations.

We need larger studies in more generalizable samples to better understand how the brain correlates of obesity map onto the cortical alterations in BD. To this end, we investigated the association between BD, medications, obesity, and neurostructural measures in a large, highly generalizable, multicenter sample from the ENIGMA-BD Working Group.

## Methods

### Participating sites

The ENIGMA-BD Working Group aims to improve replication and generalizability of neuroimaging studies of BD by combining existing, independently collected neuroimaging samples of BD from around the world (Ching et al., 2022; Hibar et al., 2018, 2016; McWhinney et al., 2021a, 2022a; Nunes et al., 2020). Seventeen independently collected ENIGMA-BD samples from 13 countries on six continents contributed individual-level structural MRI data, medication information, specifically medications used at the time of scanning for the following medication categories (lithium, first-, second-generation antipsychotics, anticonvulsants, antidepressants), and body mass index (BMI) values from a total of 1231 individuals with BD and 1601 healthy controls. Table 1 shows all participant characteristics.

**Table 1.** Demographic, diagnostic, and treatment characteristics of sample

	Controls	Cases	Significance
*N*	1601	1231	
Age, mean (s.d.)	35.47 (12.63)	42.12 (12.71)	*t*(2818) = 7.22, *p* < 0.001
BMI, mean (s.d.)	24.43 (4.12)	26.78 (5.23)	*t*(2395)^a^ = 11.48, *p* < 0.001
Normal weight/overweight/obese, *N* (%)	1014 (63.34)/437 (27.30)/150 (9.37)	509 (41.3)/436 (35.40)/286 (23.20)	χ^2^ = 163.43, df = 2, *p* < 0.001
Sex, *N* (%) female	916 (57.21)	743 (60.4)	χ^2^ = 2.71, *p* = 0.100
Diagnosis, *N* (%)			N/A
BD-I	–	904 (73.4)	
BD-II	–	318 (25.8)	
BD-NOS	–	9 (0.70)	
Treatment at time of scanning, *N* (%) / monotherapy *N* (%)			N/A
No treatment	–	218 (17.7)	
Lithium	–	556 (48.5) / 182 (46.7)	
Antiepileptic	–	425 (39.2) / 80 (22.9)	
First-generation antipsychotic	–	83 (7.6) / 5 (1.4)	
Second-generation antipsychotic	–	371 (33.9) / 53 (14.9)	
Antidepressant	–	431 (39.3) / 71 (19.9)	
Mood state, *N* (%)			N/A
Euthymic	–	598 (58.5)	
Depressed	–	365 (35.7)	
Manic	–	43 (4.2)	
Hypomanic	–	10 (1.0)	
Mixed	–	6 (0.6)	
Age of onset, mean (s.d.)	–	25.68 (10.94)	N/A
History of psychosis, *N* (%)	–	457 (52.5)	N/A

a There were no missing age or BMI values. We used the Welch two-sample *t* test (unequal variance assumed), which relies on a Welch–Satterthwaite degrees of freedom adjustment, resulting in varying degrees of freedom.

Online Supplementary Tables S1 and S2 list the demographic/clinical details for each cohort. Online Supplementary Table S3 provides the diagnostic instruments used to obtain diagnosis and clinical information. Online Supplementary Table S4 lists exclusion criteria for study enrolment. Briefly, all studies used standard diagnostic instruments, including Structured Clinical Interview for DSM Disorders (SCID; *N* = 12), Mini International Neuropsychiatric Interview (MINI; *N* = 2) and Diagnostic Interview for Genetic Studies (DIGS; *N* = 1). Most studies (*N* = 10) included both bipolar I (BDI) and bipolar II (BDII) disorders, six studies included only BDI and one study included only BDII participants. Substance abuse was an exclusion criterion in nine studies. Most studies did not exclude comorbidities, other than substance abuse. Consequently, the sample is a broad, ecologically valid, and generalizable representation of BD. All participating sites received approval from local ethics committees, and all participants provided written informed consent. The authors assert that all procedures contributing to this work comply with the ethical standards of the relevant national and institutional committees on human experimentation and with the Helsinki Declaration of 1975, as revised in 2008.

### Data acquisition and parcellation

High-resolution T1-weighted brain anatomical MRI scans were acquired at each site; see online Supplementary Table S5 for details of scan acquisition. All groups used the same ENIGMA-standardized analytical protocol, including visual and statistical quality assessment, as documented at: http://enigma.ini.usc.edu/protocols/imaging-protocols/. These protocols are standardized across the consortium, are open-source, and freely available online, in order to foster open science/replication/reproducibility. They were applied in the previous publications by our group (Hibar et al., 2018; Nunes et al., 2020) and more broadly in large-scale ENIGMA studies of major depression, schizophrenia, attention deficit hyperactivity disorder (ADHD), obsessive compulsive disorder (OCD), post traumatic stress disorder (PTSD), epilepsy, and autism (Thompson et al., 2020).

Briefly, using the freely available and extensively validated FreeSurfer software, we performed parcellations of 34 cortical regions, per hemisphere (left and right), based on the Desikan–Killiany atlas. All segmented regions were used as target regions of interest (ROIs) for analysis. We also computed total intracranial volume (ICV) to standardize surface area estimates. Visual quality controls were performed on an ROI level aided by the ENIGMA-standardized visual inspection guide including pass/fail parcellation examples. In addition, we generated diagnostic histogram plots for each site and outliers which deviated from the site mean for each structure at >3 standard deviations were flagged for further review. All ROIs failing quality inspection were withheld from subsequent analyses, see online Supplementary Table S6. Previous analyses from the ENIGMA-BD Working Group showed that scanner field strength, voxel volume, and the version of FreeSurfer used for parcellation did not significantly influence the effect size estimates.

### Statistical modeling

In this mega-analysis, we used linear mixed modeling (package *nlme* version 3.1-152 in *R* version 4.1.1) with individual subject cortical thickness or cortical surface area as dependent variables and with both BMI and group (participants with BD or healthy controls) as predictors. In each case, age, sex, and hemisphere (left or right) were also included as fixed predictors. Total ICV was included as a covariate in models of cortical surface area. Models also included a random effect of hemisphere within participants and a random effect of data collection site.

We created one model per region, with each model including both hemispheres and all of the covariates described above. We used BMI as a continuous variable, which captures more variability between participants, increases sensitivity, and was the preferred approach in most previous studies (Dekkers et al., 2019). BMI was normally distributed (online Supplementary Fig. S1). We checked the normality of model residuals using QQ plots and tested for multicollinearity using the variance inflation factor (VIF, shown in online Supplementary Table S7) of all predictor variables included in modeling. Variance in regional volumes was comparable between groups.

In post hoc analyses among individuals with BD, we separately explored the statistical effects of commonly prescribed medications. As the rates of monotherapy were low in this sample (see Table 1), we studied the association between number of jointly used medication classes (zero through three, including anticonvulsants, antipsychotics, and antidepressants) and BMI or cortical thickness or surface area. In the same model, we separately estimated the effects of current Li treatment. We used the same covariates and random-effect structure as described above. Interactions between BMI and either the number of medication classes or Li prescription were included where significant. The partial effect of the number of medication classes while adjusting for BMI was also compared with its effect without adjusting for BMI, but with all other covariates and random effects remaining. The *a priori* decision to analyze the effects of Li separately was motivated by the fact that statistical effects of Li on brain measures, which tend to be positive, may cancel the statistical effects of other medications such as antipsychotics, anticonvulsants, which tend to be negative (Hajek et al., 2012a; Hajek, Kopecek, Hoschl, & Alda, 2012b).

We adjusted all *p* values for multiple comparisons using false discovery rate (FDR), with adjusted *p* values reported, at *α* = 0.05. We calculated effect sizes for between-group differences (partial *d*), and associations between BMI and ROI volumes (partial *r*), together with their 95% confidence intervals (CIs), using model coefficients and their standard error (s.e.) (Nakagawa & Cuthill, 2007).

### Mediation analysis

We tested whether the variance in regional thickness that was associated with the number of jointly used medication classes (zero through three, including anticonvulsants, antipsychotics, and antidepressants; direct path) remained significant after also accounting for variance associated with BMI (indirect path) in individuals with BD. The number of medication classes was modeled as the associated variable, BMI as the mediating variable, and cortical thickness was the dependent variable. We modeled the direct effect of the number of jointly used medication classes on thickness, in comparison with the indirect effect of this association through BMI as a mediator, corrected for age, sex, prescription of Li, and random effects. To test this, we built 5000 bootstrapped models using random selection with replacement. This method non-parametrically identified the 95% CI for effect sizes. The bootstrap CI, which did not include zero, indicated a significant indirect effect. Simulation research indicates that the bootstrap method is more robust to non-normality and has better type I error control than the Sobel test (Hayes, 2009). Nevertheless, for methodological consistency, we also applied the Sobel test to investigate whether accounting for BMI significantly mitigated group-related differences in thickness. All of these analyses were performed in *R* version (4.1.1).

These analyses were applied only to regions which met the criteria for mediation: (1) the number of medication classes was a significant predictor of the ROI thickness, and (2) the number of medication classes was a significant predictor of the mediator (BMI), and when modelled jointly, (3) BMI was a significant predictor of the thickness, and (4) the strength of the coefficient of the previously significant independent variable (number of medication classes) was reduced. These criteria applied to the fusiform gyrus.

## Results

### Regional morphometric differences by diagnosis and BMI

**Fig. 1.**
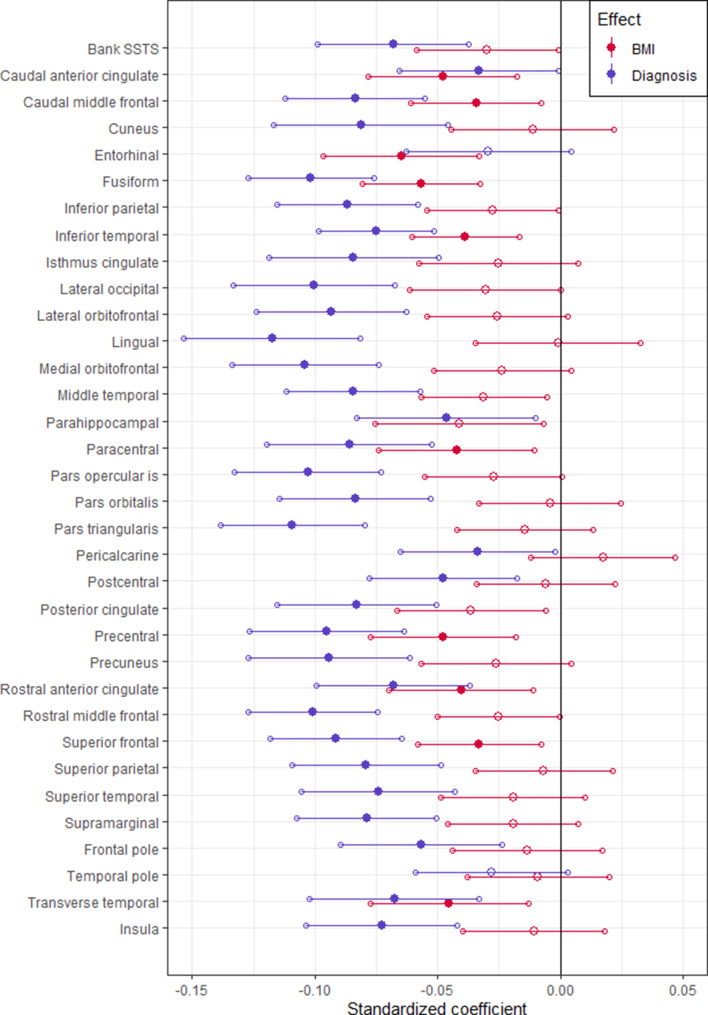
Standardized coefficients for group differences (blue) and BMI effects (red) in predicting the cortical thickness of each region. Significant effects are shown using a filled marker (FDR-adjusted *p* < 0.05).

When modeled jointly, numerous regions showed significant partial effects of either BMI, diagnosis, or both (Fig. 1, Table 2). Participants with BD showed significantly thinner cortex relative to controls in all regions except for the entorhinal cortex and temporal pole. Higher BMI was associated with thinner cortex in nine of the same regions as BD, and it was uniquely associated with thinner entorhinal cortex. Surface area did not significantly differ between groups in any region, while higher BMI was associated with larger surface area in the isthmus of the cingulate gyrus (online Supplementary Table S8).

**Table 2.** Results of multiple regression analysis in cortical thickness, including effect sizes for between-group differences (Cohen's *d*), 95% confidence interval, BMI effect sizes (part *r*), and their FDR-adjusted *p* values

			Diagnosis				BMI	
Region	Controls	Patients	Effect (*d*)	95% Low	95% High	*p*	Part *r*	*p*
Bank SSTS	1452	809	0.191	0.105	0.277	<0.001*	−0.042	0.100
Caudal anterior cingulate	1568	833	0.086	0.002	0.170	0.048*	−0.063	0.014*
Caudal middle frontal	1586	834	0.248	0.163	0.332	<0.001*	−0.051	0.039*
Cuneus	1519	820	0.196	0.111	0.281	<0.001*	−0.014	0.596
Entorhinal	1393	811	0.075	−0.011	0.162	0.089	−0.085	0.001*
Fusiform	1587	830	0.335	0.251	0.420	<0.001*	−0.094	0.000*
Inferior parietal	1565	832	0.256	0.172	0.340	<0.001*	−0.041	0.100
Inferior temporal	1559	814	0.272	0.187	0.357	<0.001*	−0.071	0.006*
Isthmus cingulate	1579	830	0.206	0.121	0.290	<0.001*	−0.031	0.199
Lateral occipital	1585	825	0.258	0.174	0.343	<0.001*	−0.040	0.103
Lateral orbitofrontal	1592	833	0.257	0.173	0.341	<0.001*	−0.036	0.140
Lingual	1576	827	0.276	0.192	0.361	<0.001*	−0.001	0.957
Medial orbitofrontal	1570	832	0.294	0.210	0.379	<0.001*	−0.034	0.160
Middle temporal	1508	817	0.264	0.178	0.349	<0.001*	−0.050	0.050
Parahippocampal	1592	833	0.107	0.023	0.191	0.014*	−0.048	0.050
Paracentral	1591	834	0.214	0.130	0.298	<0.001*	−0.053	0.037*
Pars opercularis	1571	834	0.290	0.206	0.375	<0.001*	−0.039	0.107
Pars orbitalis	1588	833	0.228	0.144	0.312	<0.001*	−0.006	0.808
Pars triangularis	1573	833	0.312	0.227	0.396	<0.001*	−0.021	0.408
Pericalcarine	1546	807	0.091	0.006	0.176	0.041*	0.024	0.334
Postcentral	1554	832	0.134	0.050	0.219	0.002*	−0.009	0.717
Posterior cingulate	1588	831	0.215	0.131	0.299	<0.001*	−0.048	0.050
Precentral	1568	832	0.254	0.170	0.338	<0.001*	−0.065	0.013*
Precuneus	1583	830	0.243	0.158	0.327	<0.001*	−0.034	0.158
Rostral anterior cingulate	1536	830	0.185	0.100	0.269	<0.001*	−0.056	0.034*
Rostral middle frontal	1582	834	0.321	0.237	0.405	<0.001*	−0.041	0.100
Superior frontal	1577	834	0.287	0.203	0.371	<0.001*	−0.053	0.038*
Superior parietal	1581	831	0.221	0.137	0.305	<0.001*	−0.009	0.708
Superior temporal	1438	815	0.205	0.119	0.292	<0.001*	−0.027	0.286
Supramarginal	1505	826	0.237	0.152	0.322	<0.001*	−0.029	0.235
Frontal pole	1584	832	0.145	0.061	0.229	0.001*	−0.017	0.495
Temporal pole	1581	835	0.076	−0.008	0.160	0.080	−0.012	0.617
Transverse temporal	1595	833	0.165	0.081	0.248	<0.001*	−0.056	0.034*
Insula	1489	830	0.202	0.117	0.287	<0.001*	−0.015	0.568

Significance is shown using asterisks (*p < 0.05)

BMI and group significantly interacted in lateral occipital cortical thickness (online Supplementary Fig. S2), with control participants showing a significant negative association between BMI and cortical thickness [*t*(2391) = −3.03, *p* = 0.002], while no significant association was seen in those with BD [*t*(2391) = 0.31, *p* = 0.757]. There was no interaction between BMI and sex for any of the regions or any of the measures. The full list of interactions is shown in online Supplementary Table S9.

### Medications, clinical variables, BMI, and brain structure

In individuals with BD, higher BMI was associated with greater number of jointly used medication classes (i.e. anticonvulsant, antipsychotic, and/or antidepressant medications) per participant [*t*(1100) = 4.89, *p* < 0.001], but not with Li treatment [*t*(736) = −0.42, *p* = 0.676].

The number of medication classes was significantly associated with smaller cortical thickness in 22 of 34 regions (64.7%), and in nearly all instances, these associations remained significant when controlling for the effects of BMI (online Supplementary Table S10). Exceptions included the pars opercularis, superior temporal gyrus, and supramarginal gyrus. There was a significant interaction between BMI and the number of medication classes in the isthmus cingulate [*t*(1858) = −4.34, *p* = 0.001], with progressively steeper associations between BMI and thickness in those with more medications (see online Supplementary Fig. S3).

There was an interaction between current Li-use and BMI in 13 of the ROIs (38.2%), including caudal and rostral anterior cingulate, medial orbitofrontal gyrus, postcentral gyrus, pars opercularis, pars triangularis, rostral and caudal middle frontal, superior frontal, superior temporal, supramarginal, frontal pole, and insula, such that people who were prescribed Li at the time of scanning showed stronger negative association between BMI and cortical thickness than individuals with BD who were not treated with Li (online Supplementary Table S10 and Fig. S4). Amongst the remaining regions, which did not show interaction between BMI and Li, Li was positively associated with cortical thickness even when controlling for the negative effect of BMI in nine regions, including cuneus, precuneus, inferior parietal, lateral occipital, lingual, paracentral, precentral, pericalcarine, and superior parietal gyri.

BMI, Li treatment, and the number of medication classes showed negligible multicollinearity (VIF < 1.004). BMI was not significantly associated with illness duration, history of psychotic symptoms, diagnostic subtype, or mood state (online Supplementary Table S11).

### Mediating effect of medications

**Fig. 2.**
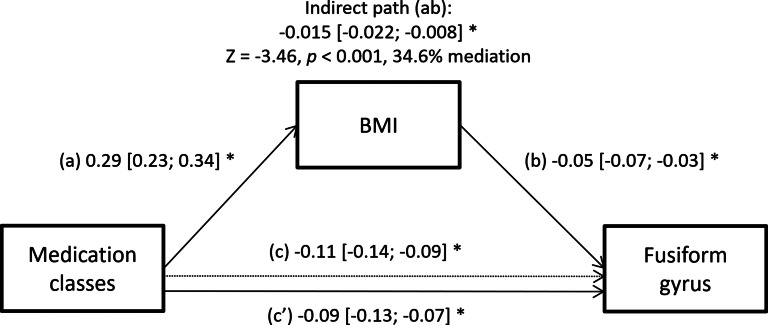
The effect of medication classes and BMI on cortical thickness. Path (c) represents the direct effect, while (a) through (b) represent the indirect path through BMI, and (c′) represents the adjusted direct effect after accounting for BMI. We show standardized coefficients along with their 95% CI derived from bootstrapping. Significant effects (95% CI that excludes zero) are marked by asterisks. In all models, we controlled for the covariates age, sex, Li treatment, and data collection site, while paths b, c, and c’ were additionally adjusted for a random effect of hemisphere.

Only the fusiform gyrus met the criteria for investigating whether BMI mediates the relationship between the number of medication classes and cortical thickness (online Supplementary Table S10). Specifically, there was a significant indirect effect of the number of medication classes on lower fusiform gyrus thickness through BMI (Est = −0.015, 95% CI −0.022 to −0.008), with 34.6% mediation (*Z* = 3.46, *p* < 0.001, see Fig. 2).

## Discussion

In this study of 2832 individuals, we found substantial overlap between regions associated with BMI and BD. Specifically, with exception of a single ROI (entorhinal cortex), all of the regions which were negatively associated with BMI were also negatively associated with the diagnosis of BD. In contrast, only a single ROI (isthmus of the cingulate gyrus) showed association between BMI and surface area, which was positive. Importantly, about a third of the negative association between number of psychiatric medications and cortical thickness in the fusiform gyrus was partially mediated by the association between number of medications and higher BMI. Furthermore, the association between BMI and cortical thickness in isthmus cingulate became steeper with higher number of medication classes used jointly. In contrast, significant negative associations between BMI and cortical thickness in isthmus cingulate and rostral anterior cingulate became non-significant when modelled jointly with a significant positive effect of Li on cortical thickness. There was an interaction between Li and BMI, such that the brain correlates of BMI were more pronounced in individuals with *v.* without current Li treatment in a number of frontal regions, as well as anterior cingulate. The statistical effect of BMI on brain structure was linear in all regions, thus it would be most pronounced in people with obesity, but also manifest in overweight individuals.

The cortical correlates of obesity in this study closely replicated findings from previous large-scale studies, which also reported negative associations between obesity and cortical thickness or volume, especially in caudal and rostral anterior cingulate, entorhinal cortex, and several frontal lobe regions (Janowitz et al., 2015; McWhinney et al., 2022b; Opel et al., 2021), but with much fewer correlates in surface area (McWhinney et al., 2022b; Opel et al., 2021). These are some of the same regions which are consistently associated with BD, but also with other major psychiatric disorders (Hibar et al., 2018; van Erp et al., 2018). Interestingly, in all of these regions both BMI and BD showed partial association with cortical thickness, when controlling for the other factor. In other words, brain changes in cingulate and frontal regions will be greater in people with both obesity and BD than in those with either condition alone.

It is unclear whether these regions are particularly susceptible to obesity, or whether their changes predispose individuals to obesity. The mechanisms through which brain structure could predispose to obesity involve alterations in the reward system (Opel et al., 2015), cue triggered learning and Pavlovian conditioning to hedonic food (Meyer, Risbrough, Liang, & Boutelle, 2015), and in appetitive behavior (Löscher, Brandt, & Ebert, 2003; Malkova, Mishkin, Suomi, & Bachevalier, 2010). On the other hand, obesity could affect brain structure through a range of mechanisms, including among others systemic inflammation, oxidative stress, insulin resistance/diabetes, hypertension or dyslipidemia (Cox et al., 2019; Goldstein et al., 2020; Hajek et al., 2014; Parimisetty et al., 2016; Van Gaal, Mertens, & De Block, 2006; Willette & Kapogiannis, 2015; Wisse, 2004). Speculating about how the regions described above could predispose to obesity would be post-hoc and inconclusive. However, previous studies investigating associations between genetic risk for obesity and brain structure reported involvement of a much smaller set of regions, i.e. surface area of lateral occipital lobe (Opel et al., 2021) or medial prefrontal volume (Opel et al., 2017) than those reported here and in other previous studies of association between obesity and brain structure (Janowitz et al., 2015; McWhinney et al., 2022b; Opel et al., 2021). Therefore, brain correlates of obesity most likely include both causes and consequences of obesity. Considering the greater extent of associations between obesity and brain structure relative to the cih the much smaller extent of associations between genetic risk for obesity and brain structure, most brain changes likely represent consequences of obesity.

An especially interesting and relevant question is the role of obesity in brain effects of psychiatric medications. In keeping with other studies, we found that antipsychotics and anticonvulsants were negatively associated with brain structure (Andreasen, Liu, Ziebell, Vora, & Ho, 2013; Fleisher et al., 2011; Fusar-Poli et al., 2013; Hibar et al., 2016, 2018; Tariot et al., 2011; Van Gestel et al., 2019) and positively with BMI (Mitchell et al., 2013; Tek et al., 2016). Considering the negative association between obesity and brain structure, perhaps the negative associations between medications and brain structure could be mediated by obesity (Joober, Schmitz, Malla, Sengupta, & Karma, 2006; McWhinney et al., 2021a, 2021b). Across most regions, the association between number of medications and cortical thickness remained significant when we controlled for BMI, which is in keeping with another study (Jorgensen et al., 2017). In a single region, the fusiform gyrus, about a third of the association between medications and cortical thickness was related to the indirect association between medications and BMI and between BMI and brain structure. In a single region, isthmus cingulate, the association between BMI and cortical thickness became steeper with growing numbers of medications used jointly. So, while the obesitogenic effects of medications may mediate or moderate the negative association between medications and brain structure in some regions, for the most part, the negative statistical effect of medications on brain structure was independent from their obesitogenic effects.

A separate question is the interplay between the putative neuroprotective effects of Li and its impact on weight. Interestingly, in this study, Li remained positively associated with cortical thickness across numerous regions, even when we controlled for negative associations between BMI and regional thickness. Conversely, a significant negative association between BMI and cortical thickness became non-significant in the isthmus cingulate and rostral anterior cingulate when modelled jointly with the significant positive effect of Li on cortical thickness. In other words, BMI did not cancel the positive association between Li and cortical thickness, while Li did cancel the negative association between BMI and cortical thickness for some regions.

Interestingly, in some regions, people who were using Li at the time of scanning showed stronger negative association between BMI and cortical thickness than BD individuals not treated with Li. These findings were quite robust and replicated across approximately one-third of regions. It is possible that regions which are negatively associated with BD will not show negative effects of additional variables, such as BMI, unless the impact of the illness is mitigated by, for example, Li. Indeed, we found this interaction mostly in regions which were more strongly associated with diagnosis than with BMI, including medial orbitofrontal gyrus as well as pars opercularis and triangularis of the inferior frontal gyrus. In addition, this interaction among individuals with BD could have decreased the apparent effect of BMI in these regions. Indeed, in the whole group, BMI was not associated with thickness of these regions. This is also in keeping with greater negative association between BMI and cortical thickness in controls than in people with BD.

Since we do not understand the origins of brain alterations in BD, it is highly relevant to study variables which could be associated with the structure of the brain, such as BMI. On the theoretical level, such studies could help explain the differences in brain measures among people with the same diagnosis, as they may differ in BMI. They could also explain similarities in brain measures across people with BD or schizophrenia, as they share a high risk of obesity. On the clinical level, comorbid obesity in people with major psychiatric disorders is associated with poor functioning, greater risk of chronicity, disability and suicide, poor treatment response, and functional deterioration (Bora, Akdede, & Alptekin, 2017; Calkin et al., 2015, 2009; Hajek et al., 2008, 2005; McIntyre et al., 2010; Salvi et al., 2020). The associations between obesity and brain structure might help explain these links and provide new treatment options for some of these currently difficult to treat outcomes. Lastly, similar studies could help identify risk factors for neuroimaging outcomes, which may provide new opportunities for prevention or treatment of brain alterations with dietary/lifestyle medication or surgical interventions focused on weight management (Mansur et al., 2017; Mueller et al., 2015; Shan et al., 2019; Tuulari et al., 2016).

This study benefits from several unique advantages. The large sample size (2832 individuals) allowed us to test for interactions among relevant factors, which could not be conclusively studied in smaller, less powered studies. The multi-site nature of this study, with data from 17 sites in 13 countries, ensured highly generalizable representation of BD from around the world. We focused on overweight/obesity, which is a highly prevalent, but understudied factor in relation to brain structure in BD and which could also provide important insights into negative associations between obesitogenic medications and brain structure. In addition to novel findings on the interplay between BMI, BD, or medications and brain structure, we provide several replications of previous findings of associations between obesity or BD and specific regions of interest.

This study has the following limitations. The cross-sectional nature of our study does not allow us to discern the direction of the association, as brain alterations may predate or result from obesity. More detailed markers beyond BMI were not broadly available throughout the ENIGMA-BD Working Group. At the same time, BMI is much easier to acquire and is by far the most frequently used measure (García-García et al., 2019; Willette & Kapogiannis, 2015), thus allowing for a more direct comparison with previous studies. Due to confidentiality reasons related to legacy datasets, we could not access raw, whole-brain data and could not use methods, such as voxel-based morphometry. Aside from using ENIGMA-standardized processing methods, we also addressed any differences between scanners statistically by using mixed models and including site as a random factor in all analyses. While there are other approaches, this is still by far the most utilized and accepted method for dealing with site effects (McWhinney et al., 2021a; Thompson et al., 2020). Information about medications was limited to current usage at the time of scan. The study was not designed to comprehensively test the effects of medication, which would require a randomized controlled design. Therefore, the medication findings should be interpreted with caution, as medication prescriptions in clinical practice are not random. Also, medication details were limited to current prescription, without any measures of duration, dosage, compliance, previous medication exposure, treatment response or symptom levels at the time of prescription, so we cannot address the effects of these factors. The basic ENIGMA covariates, which are available across sites, did not contain any measures of cognitive/psychosocial functioning. Therefore, we could not evaluate the structure/function links. Fat content near the MRI coil may lead to slight signal intensity changes, but the vast majority of individuals in this study were normal weight to overweight. Psychiatric and other medical comorbidities, which might not be available for all the patients enrolled, may influence the interplay between BMI, BD, and neuroimaging findings. Finally, using other neuroimaging modalities could provide further insights into the mechanisms of the BMI effect. Last but not least, caution is needed when interpreting mediation analyses in observational studies.

To conclude, we confirmed consistent associations between higher BMI and lower cortical thickness across the cerebral mantle, in regions which were also associated with BD. There were few or no correlates of either condition with cortical surface area. In most regions number of medications remained associated with lower cortical thickness regardless of BMI, but there were also instances of mediation and moderation of associations between number of medication classes and cortical thickness by BMI. In terms of Li treatment, either the positive association between Li and cortical thickness was present regardless of BMI or people treated with Li showed steeper association between BMI and cortical thickness. All in all, BMI is important for understanding the neuroanatomical alterations in BD and the neurostructural correlates of psychiatric medications. We need prospective studies to investigate whether obesity is a modifiable/preventable risk factor for brain alterations in BD and whether the obesity-related negative psychiatric outcomes are related to obesity-related brain alterations.

## Supplementary material

For supplementary material accompanying this paper visit https://doi.org/10.1017/S0033291723000223. click here to view supplementary material
